# Nature Prescriptions and Indigenous Peoples: A Qualitative Inquiry in the Northwest Territories, Canada

**DOI:** 10.3390/ijerph21060806

**Published:** 2024-06-20

**Authors:** Nicole Redvers, Jamie Hartmann-Boyce, Sarah Tonkin-Crine

**Affiliations:** 1 Schulich School of Medicine and Dentistry, University of Western Ontario, London, ON N6G 2M1, Canada; 2 Department for Continuing Education, University of Oxford, Oxford OX1 2JA, UK; 3 Nuffield Department of Primary Care Health Sciences, University of Oxford, Oxford OX2 6GG, UK; jhartmannboy@umass.edu (J.H.-B.); sarah.tonkin-crine@phc.ox.ac.uk (S.T.-C.); 4 Department of Health Policy and Promotion, University of Massachusetts Amherst, Amherst, MA 01003, USA

**Keywords:** Indigenous Peoples, nature prescription, nature prescribing, park prescriptions, green prescribing, green prescription, Northwest Territories, qualitative research

## Abstract

Nature prescription programs have become more common within healthcare settings. Despite the health benefits of being in nature, nature prescriptions within the context of Indigenous Peoples have received little attention. We therefore sought to answer the following question: What are circumpolar-based physicians’ and Indigenous Elders’ views on nature prescribing in the Northwest Territories, Canada? We carried out thirteen semi-structured interviews with physicians between May 2022 and March 2023, and one sharing circle with Indigenous Elders in February 2023. Separate reflexive thematic analysis was carried out to generate key themes through inductive coding of the data. The main themes identified from the physician interviews included the importance of cultural context; barriers with nature prescriptions in the region; and the potential for nature prescriptions in the North. Reflections shared by the Elders included the need for things to be done in the right way; the sentiment that the Land is not just an experience but a way of life; and the importance of traditional food as a connection with Nature. With expanding nature prescription programs, key considerations are needed when serving Indigenous communities. Further investigation is warranted to ensure that nature prescriptions are appropriate within a given context, are inclusive of supporting Land-based approaches to health and wellbeing, and are considered within the context of Indigenous self-determination.

## 1. Introduction

The health benefits of being within nature or having exposure to greenspaces have been increasingly described. Those who spend time in nature [1] or have access to greenspace [2] report better health and psychological wellbeing compared to those who do not, with the effects being crosscutting across different occupations, ethnic groups, rich and poor areas, and in people with chronic illnesses and disabilities [1]. Physiological stress reduction [3,4], improved cardiovascular health [5,6], memory improvements [7], increased natural killer cell activity [8], and increased longevity in senior citizens [9] are benefits associated with spending time in or near nature.

In recognition of the noted health benefits, organized national park and nature prescription programs have increasingly been developed within Canada and abroad [10,11]. A park prescription program called PaRx, for example, is breaking ground as Canada’s first national, evidence-based nature prescription program with several regional and national partners [10]. Park prescription interventions (PPIs) have generally demonstrated improved physical activity in parks, recreational physical activity, and improvement in psychological quality of life [12]. A recent systematic review and meta-analysis also found that by incorporating nature-based social prescription interventions into mental healthcare plans, improved mental health outcomes occurred [13]. Additionally, nature prescriptions fit under the umbrella of “patient–planetary health co-benefit prescribing” [14], with research not only demonstrating the health benefits for patients but additionally the planet, with spending time in nature being associated with increased pro-environmental attitudes and behaviours [15]. Given the increased attention to nature prescriptions as a therapeutic intervention, there is a need to ensure the consideration of programs such as PaRx are examined through a culturally safe lens with populations that may have long-standing relationships with nature.

For example, despite the perceived and demonstrated health benefits of being in nature, nature prescriptions themselves within the context of Indigenous Peoples in Canada and globally have received little attention. As Indigenous Peoples have lived in close relationship with the planet for millennia, the context of having Western health professionals prescribing nature is not often considered in current nature prescription programs. Indigenous-specific nature programs have been increasingly platformed from within Indigenous communities in Canada; however, they have been most often situated within the context of Land-based healing, cultural, and education programs, and not necessarily within healthcare settings [16,17,18,19]. Given this, we sought to listen to the perspectives of physicians working with a high proportion of Indigenous patients, as well as a group of Indigenous Elders within the Northwest Territories (NWT) on “patient–planetary health co-benefit prescribing” [20]. In this current study, we sought to analyze data specifically on nature prescriptions within the context of Indigenous patient care. We asked, what are circumpolar-based physicians’ and Indigenous Elders’ views on nature prescribing in the NWT?

### Positionality

Any research involving Indigenous Peoples increasingly demands that authors position themselves in relation to the work [21,22]. NR is a member of the Deninu K’ue First Nation located within the NWT and an Indigenous health scholar, JHB is a health policy researcher based in the United States, and STC is a health researcher based in the United Kingdom studying prescribing behaviour in primary care.

## 2. Methods

We originally designed two pragmatically orientated [23] qualitative research studies allowing space for a decolonized research paradigm (i.e., embedding a “collaborative process of naturalizing Indigenous intent, interactions, and processes and making them evident to transform spaces, places, and hearts” [24]) on patient–planetary health co-benefit prescribing with physicians (semi-structured interviews) [20] and with Indigenous Elders (sharing circle) [25] in the NWT. Our pragmatic approach allowed us to be solutions-orientated and action-based [23]. In speaking to two locally based physicians and Indigenous community members (including an Elder) based in the NWT prior to the onset of these primary studies, feedback was given to us on the perceived gaps in the understanding of nature prescriptions in the context of Indigenous Peoples in the region. This discussion was prompted by the potential expansion of the PaRx into the NWT. Given the importance of being responsive to the needs of the region, we identified a unique research question and proceeded to add questions to our interview and sharing circle topic guides specific to nature prescribing within our primary studies and carried out a separate data analysis, which we share here. Standards for reporting qualitative research (SRQR) [26] were followed, and we gained ethics approval from (1) the Departmental Research Ethics Committee at the University of Oxford (#SSHEQ_C1A_21_023) and (2) the Aurora Research Institute based in the NWT (NWT research license #16938).

### 2.1. Setting

The research was carried out in the Northwest Territories (NWT), Canada, which is located in the northern part of the country and has a majority Indigenous population (i.e., First Nations, Inuit, Métis Peoples). There are eleven official languages in the region, including Dene Kǝdǝ́, Dëne Sųłıné, Dene Zhatıé, Dinjii Zhu’ Ginjik, English, French, Inuinnaqtun, Inuktitut, Inuvialuktun, nēhiyawēwin, and Tłı̨chǫ. The geographic area is vast (land mass of 1,171,918 square km [27]) despite only having a total population of approximately 45,000 people. Physicians who are based in the NWT are usually located either in Yellowknife (the capital city) or less commonly in the smaller community centres due to ongoing physician shortages in the area [20].

### 2.2. Recruitment and Consent

Purposive sampling [28] was used in both primary studies to ensure maximum variation in the physician participants (*n* = 13) with regard to gender, years of practice, specialty, and the practice region within the NWT; for the Elders (*n* = 6), maximum variation was ensured with regard to region within the NWT and gender. We do not report specific participant characteristics due to the likelihood of of participants being identified within a very small region. Recruitment occurred through existing networks and contacts known to the lead author (NR). A record of consent was taken verbally after the participants received emailed information on this study, had the opportunity to ask any questions they might have on this study, and agreed to participate. Honorariums were provided to the Elders for participation.

### 2.3. Interview and Sharing Circle Data Collection

Between 16 May 2022 and 10 March 2023, thirteen semi-structured interviews that lasted up to an hour were carried out and audio-recorded virtually with the physician participants. On 28 February 2023, the sharing circle with Indigenous Elder participants was carried out virtually and audio-recorded. Our data were collected virtually as the original approvals for the research were received when COVID restrictions were still in place. Additionally, given the vast geographic spread of the region, having the research take place virtually allowed for greater participation across the remote regions. Analytic memos were taken alongside both the interviews and the sharing circle with regular debriefs being held with the research team throughout the data collection phases to facilitate an ongoing reflexive practice. Both the interviews and the sharing circle followed topic guides with open-ended questions that allowed for exploration of the topic area openly and without restriction (see Supplementary Materials). Key topics outlined in the topic guides were informed by the literature as well as through engagement with two local physicians and two Indigenous Elders to better ensure the questions were clear, understandable, and relevant. The interviews and sharing circle were carried out in English, and we queried on topics including perspectives around giving or receiving nature prescriptions, situations where nature prescriptions might be helpful or where there might be challenges in their delivery, as well as the perceptions around the terminology framing of ‘nature prescriptions’ (see Supplementary Materials).

### 2.4. Data Analysis

All audio recordings were transcribed verbatim and were then sent to all individual participants to ensure their comfort was met with the level of anonymization given the smaller region and higher chance of certain details identifying someone (e.g., only one known physician works in a specified community). The interview and sharing circle transcripts were loaded into NVivo qualitative software (Release 1.3) for data analysis; however, the physician interviews were analyzed separately from the Elders’ sharing circle to ensure the varied worldviews could be appropriately respected and not assimilated within the analysis. Additionally, for the work with both the physicians and the Elders, we analyzed only the data within the wider transcripts that were relevant to our nature prescription research question. Reflexive thematic analysis was carried out as outlined by Braun and Clark (2006) [29] and advanced in 2019 [30] to generate key themes through inductive coding. Coding stages were tracked through the use of analytic folders to keep an audit trail. One of the authors carried out the coding (NR) with a second author brought in for discussion and for the refining of the codes and themes (ST-C). We referred back to the original transcripts repeatedly to support the refining of codes and themes.

## 3. Results

### 3.1. Physician-Level Perspectives

There were three main themes identified from the physician interviews reported below. The physicians highlighted key elements for consideration when it comes to nature prescriptions within the NWT region, including cultural considerations and the positionality of non-Indigenous physicians in relation to Indigenous patients with regard to nature prescriptions; the need to be mindful of the winter climate and how this might affect the nature recommendations given; terminology considerations that may better fit with Indigenous worldviews of interacting with nature; and widening the interpretation of what a nature prescription could look like in practice.

#### 3.1.1. The Cultural Context Matters for Considering Nature Prescriptions with Indigenous Peoples

Physicians noted the importance of the cultural context when considering nature prescriptions in the NWT given the colonial history. For example, the *framing* of nature prescriptions was repeatedly said to matter given the higher Indigenous population in the NWT and the high proportion of non-Indigenous health providers. There was a noted risk that if nature prescriptions were not carried out in the right way, it could break trust with Indigenous patients, and also be seen as paternalistic—especially in the context of smaller and more remote Indigenous communities.

*Well, you have to contextualize. I’m saying this to some Indigenous Peoples, like who the hell am I? The cultural context I think is important. That may be a challenge it may be perceived as paternalistic and sort of contrary to our shared history, when in fact … and as a non-Indigenous individual who’s been part of this process of, how do you say it, not exactly promoting the destruction of nature but I think you understand what I’m saying*.

(ID 1.6)

With this, the use of *prescribing* in the context of nature was noted to be a problematic term, with a level of cultural inappropriateness when presented in a way that is directive towards Indigenous Peoples given the power differentials inherent in colonial systems of healthcare (e.g., non-Indigenous physician prescribing a nature treatment to an Indigenous patient whose culture is inherently connected to nature).

*When I’m working in a primarily Indigenous context, I am a visiting settler, I’m very aware of that. I feel absolutely not comfortable, and I feel like it’s inappropriate to talk about prescribing nature and I think we’ve had those discussions in our meetings before about how challenging that is, to have something that’s been part of culture and a lifestyle forever, then being medicalized and written on a piece of paper, like it’s so ridiculous*.

(ID 2.1)

*… to prescribe nature to an Indigenous person. We might have to have a conversation about that. We probably should, now that I think about it actually, but before we launch that program, or at least really look at the messaging, to make sure that the messages are communicated in a non-patriarchal way that acknowledges that we are now a western entity that is recommending a return to what was and is a way of life*.

(ID 3.7)

One physician noted that nature prescriptions might be better received from Indigenous health providers.

*But I think just in the overall context of prescribing nature or time on-the-Land or all that, I think if you have healthcare providers here, nurses, doctors, other providers, who are Indigenous or who are invested long term in the territory, you have that type of longitudinal connection with the patients that those types of initiatives [Nature prescribing] can be better received. I think we’re not in a situation to have that in the territory right now* (ID 2.4). Another physician noted that, given the cultural considerations, they would feel comfortable giving nature prescriptions only if the request for incorporating this kind of approach came from Indigenous community voices.

*To me I would only be on-board with that [nature prescriptions] if it was coming from within a [Indigenous] community, that they were hoping to kind of implement that within their community, and I’d be happy to jump on-board, but I wouldn’t be wanting to invoke something like that that wasn’t coming from within the community [given the cultural considerations], that wasn’t something they’d decided was a priority for their community members*.

(ID 1.2)

Ultimately, there was an appreciation for the sensitivities inherent in a context where often Westernized health professionals are caring for Indigenous Peoples who have had deep relationships with the Land for millennia. Therefore, there was an understanding of the need to be carefully attuned to the perceptions Indigenous Peoples might have regarding nature prescriptions within the clinical environment.

#### 3.1.2. Highlighted Barriers with Nature prescriptions in the NWT Region

There were many key barriers noted for nature prescriptions in the North that were thought to be in need of consideration. One barrier that was also directly interconnected to the cultural context (i.e., the cultural context matters) was framed from the following question: Is it actually culturally appropriate for me to give a nature prescription as a settler physician to an Indigenous person? Many of the physician participants noted a clear level of discomfort with the idea of prescribing nature to their Indigenous patients. This discomfort was in addition to the need for a general understanding of the cultural context of the region.

*Telling Indigenous patients that they need to spend more time on the land, being a white person in a white institution that has done a number of horrific things in the past and continues to do so, is very problematic, and I’m very sensitive to that. So, knowing what’s right for the patient and giving an order, or a prescription, for the patient, to do something that was taken away from their people by the people of the doctor, I’m not comfortable with*.

(ID 1.5)

Other physicians were less sure whether or not a nature prescription would be welcomed.

*I would wonder, from an individual standpoint as a white [gender], if I would be welcomed to provide something like a park prescription to an Indigenous person, if that would be received well or poorly, and there would probably be a lot of variables, including the context in which we were interacting and our rapport and things like that*.

(ID 2.5)

In some cases, the concern around Settler to Indigenous relations in the context of nature prescribing was to the extent that the physician would feel comfortable offering this in the *south* of Canada but not in the context of the *north* of Canada given the higher proportion of Indigenous Peoples in the North. Nature prescriptions were noted to be a *bit odd* in the context of the North.

*I think if I was in a bigger city [in the South], I might be more open to that, especially if there are people who never leave their apartment or only working in the city and never really getting out. But I think living up here [in the North], and certainly I would never, ever say that to somebody who’s lived here for generations, who has their own relationship to the land. I’d feel it would be inappropriate for me to say that and kind of paternalistic, frankly*.

(ID 1.2)

Another barrier to nature prescribing noted in the NWT was cold weather and the ability to afford clothing for cold weather.

*But still even in the winter, we get lots of people who hunker down in their apartments all winter long and get really, really depressed. So, I think the barrier is simply environmental, three hours or no hours of daylight, with temperatures that can frequently be stuck somewhere between −35° and −45°, is a pretty big barrier for some people sometimes*.

(ID 2.2)

*I’m cautious again about making that outdoor prescription, or nature prescription, especially in the middle of winter, because if people don’t have the wherewithal, or even the clothes, like the expense or the amount of money it costs to get the right clothes to be able to spend time outside. That’s also a factor. So, if you’re prescribing a family that’s living on $500 a month, you need to take your kids outside more often throughout the winter months, that’s just pushing them further and further away from trusting you as a medical professional and actually taking your advice*.

(ID 1.5)

The dark winters in the North and other safety considerations (e.g., environmental conditions) were mentioned as considerations to take into account in the context of the region.

*In the NWT, our environment is inherently dangerous. It’s beautiful, it’s wonderful, there are so many cool opportunities, and the connection to the land can be so profound, but that always comes with, you need to respect the land, you need to respect the environment, because it can swallow you whole if you’re not prepared*.

(ID 1.5)

Others mentioned that having the available time to be in nature could be a barrier as well as the ability to have access to nature, in addition to resource barriers to spending time on the land (e.g., social determinants of health).

*If somebody is impoverished and struggling just to make ends meet and raise their children, the thought of going out and spending time in nature can be a huge barrier*.

(ID 4.7)

Lastly, the high reliance on locum physicians in the North with little rapport with Indigenous patients was noted as a potential barrier, as well as potential issues with urban or intergenerational patient disconnection from being on the Land given the colonial history in the region.

#### 3.1.3. The Potential for Nature Prescriptions in the NWT Health System with More Awareness and Appropriate Communication on the Importance of Being on the Land

Despite many of the stated challenges and barriers noted within the NWT context, there were several physicians noting that there is still lots of nature potential in the North with more amplification on the ongoing importance of being on the Land, including for mental health, needed within the health system.

*Man, we can access nature within minutes of just about anywhere in the North…We’re just so fortunate to live in a part of the world where we’re absolutely blessed with an absolutely phenomenal nature environment that’s easily accessible*.

(ID 2.2)

Almost half of the physicians interviewed were already giving some form of nature prescription, but not in the context of a *written* prescription or stated as *nature prescriptions*. Several physicians noted they were giving *recommendations* to patients *verbally* instead of in Western-based prescription formats. Many physicians noted the importance of trying to *speak the language* of the people instead of using nature prescription terminology itself. For example, several physicians felt that using *on-the-Land* or *connection to Land* kinds of terminology in the context of Indigenous patient care was more comfortable for them, while still being careful that the recommendations not be directive in nature.

*I am comfortable with talking to patients, especially Indigenous patients in communities, and in [community], wherever, about the helpful impacts of being outside and being connected to the land, because I’ve seen that amongst many other patients, and friends and family. And speaking about whether that resonates with that patient and if that patient thinks that that might be helpful. But I’m very cautious and uncomfortable with giving directives, if that makes sense*.

(ID 1.5)

There was a note that prescribers likely would be on board if nature prescriptions were approached with “cultural safety” in mind (i.e., fostering “an environment where people of diverse cultural and ethnic backgrounds can feel respected and safe—spiritually, socially, emotionally, and physically—from discrimination and denial of their identity and needs” [31,32]), and an acknowledgement that the type of nature activity in the north of Canada is different from the south of Canada.

*We talk about nature differently in the North. It’s more spending time on the land and that kind of thing and I think the very prescriptive, go out and spend 30 min at least five times a week, it’s very Western, colonial medicine. So, I have always tried to tailor it in different ways, depending on who I’m talking to. So more an emphasis of spending time on the land and for a lot of people that means going out hunting and trapping and you’re spending a week on the land, as opposed to 45 min five times a week or whatever. So, I have tailored it a little bit that way*.

(ID 4.7)

Nature was noted to not be a new health discovery in the context of Indigenous Peoples, and there were many culturally relevant ways to facilitate this kind of conversation in a patient visit that was inclusive of many relevant outdoor activities.

*I think for a lot of patients it’s more relevant to say time on the land where you’re being active, you’re going outside and you’re having to chop wood or carry water or do some physical task around your camp, that kind of thing. I think that perhaps is more meaningful than something like exercise prescription. I think a similar thing for both nature prescriptions and exercise prescriptions, there’s perhaps some wording that can engage patients a bit better in different cultural settings*.

(ID 2.4)

For interacting with Indigenous patients, these other kinds of relevant outdoor activities were noted to support the introduction of a more appropriate context of being on the land for the health and wellbeing benefits of Indigenous Peoples.

*Writing it on a piece of paper, you will go outside for five minutes three times a day for the next week, I could trial it, I could make a trial of it but we end up having more holistic conversations about being outdoors. When were you on the land last? When did you go fishing last? When did you go hunting last? Who did you go with? How long were you out there for? When do you plan to go back? Would you want to go back sooner or are you okay with going there? Is there a reason why you’re not able to go sooner or go when you would like to go?*.

(ID 2.7)

There was also a clear recognition of the overall benefits of nature prescriptions—especially if combined with exercise prescriptions.

*So if the exercise is in the daylight in nature and I want you to be out in nature because of all of the evidence around changes in brain chemistry when you’re in nature and I go through that and I’m like, you can do that in three separate chunks or you can do that as basically going for a walk in the middle of the day outside and you get all of them all at once*.

(ID 4.1)

There were some additional recommendations shared for having more grand rounds (i.e., group learning activities within clinical settings) on the topic, and having a formal system in place to provide appropriate guidance for Indigenous patients.

### 3.2. Indigenous Elder-Level Perspectives

We report findings from the Elders in this section without defined categories or quote assignments in keeping with an Indigenous paradigm of sharing everything together as a whole. In the sharing circle, some of the Elders had a hard time seeing Nature (purposely capitalized in the Indigenous context to denote its significance as a living relative) as being a *prescription*; however, if this approach were to be taken, then the process in which it would be done was seen to be very important.

*…we all want to go home with a prescription, but I think it’s a good idea and I can see it being done, but the process in which it’s going to be done is important… Because I can’t see it as a prescription, although I’m a big advocate for being outdoors and walking and getting as much as I possibly can… Anyway, for me personally I truly believe in that but… I think you need an information campaign.*

It was noted that *if it’s done in the right way*, this kind of initiative may be perceived to validate Indigenous ways of traditional medicine and being on the Land.

*I think if it’s done in the right way, it could be perceived as validating what your people have been doing…is what you need and also included in that could be the traditional medicines…*

*So, I think if it’s very well done…I think it’s going to be better for ourselves…for stress and for taking a rest, and done by professionals and it’s legitimate. Even if it’s not taking us over to the pharmacy, it’s taking us to the land…*

There were some reflections about whether community-based activities might be more beneficial, such as starting a local walking group compared to an individual form of recommendation. There was also a note of potentially hiring somebody from the local community to take people out on the Land to let them rest away from the modern world (e.g., technology) as an option for this kind of initiative.

*…if the hospitals know that illness is caused by stress and they say you should rest, but resting in a foreign environment, they put you back into the same environment and say rest. But if they give a prescription to say rest, you can maybe give it to your office, with some funds from Health Canada or something to say this person needs to have a rest, it should be outdoors. If they hire somebody from the local community to take them out by boat and take them for a ride into the environment, have a campfire and set up a little tent for a couple of days and just let them rest or maybe even a cabin somewhere, so that environment is… the people are kind of treating themselves.*

There was also a clear reference to the Land not just being for a Nature experience but as *a way of life* that may not be appreciated by the health system.

*I like to go and check out the Land, the water, as I talk to them about how it is over there in our homeland when we go hunting and fishing or just going out there for a week or a couple of days. It’s not like a tourist base for us, it’s real for us. It’s a way of life for us and we have all our stories out there, the Creation stories and all our culture and language comes from there, comes from the sound of animals and then we communicate like that with each other.*

One Elder noted that maybe the physicians are the ones who need the Nature prescription.

*I think it’s going to be better for ourselves to be able to get that and we can provide that prescription to those doctors.*

Many of the Elders additionally noted that Indigenous traditional foods should also be encompassed under the umbrella of Nature prescriptions, thereby not limiting them to Western definitions of what Nature interactions should be constituted as.

*And just eating natural foods for maybe a week might make some improvement and that kind of prescription can be brought back to the community and that means that somebody has to provide the fish.*

*Maybe somebody in the community who’s a fisherman and regularly has a fishnet out there, maybe they might get a prescription to say provide this person with a fish a day and even fillet it for them and just use natural cooking over the fire, and that means that… in a hospital, they provide that stuff and every time an Elder is admitted to the hospital, the first thing they complain about is the food. If it’s natural, then they wouldn’t be complaining.*

Ultimately, a more holistic Indigenous view was said to be needed for what might constitute a nature prescription in the North.

## 4. Discussion

Physicians in our study highlighted key elements for consideration when it comes to nature prescriptions within the NWT region of Canada, including the importance of cultural context and the positionality of non-Indigenous physicians in relation to Indigenous patients with regard to nature prescriptions; the need to be mindful of the winter climate and how this might affect the nature recommendations given; terminology considerations that may better fit with Indigenous worldviews of interacting with nature; and widening the interpretation of what a nature prescription could look like in practice and within the health system. The reflections that were shared by the Indigenous Elders included the need for things to be done in the right way if such a program were to move forward; that the word *prescription* in regards to nature may not be the most appropriate framing; the view that the Land is not just an experience but is a way of life that includes culturally based activities that should be supported; and the importance of traditional food as a connection to Nature.

To the best of our knowledge, this is the first study in which nature prescriptions were examined within an Indigenous patient context. Although there are a few studies that have been carried out on green prescription ‘health outcomes’ within Indigenous Peoples in other geographic contexts [33,34], none of the findings explicitly address the inherent barriers or challenges to this kind of initiative. More specifically, there has been no examination of the context where nature prescriptions are given to people whose traditional livelihoods are or have been entirely Land-based and are receiving colonial forms of healthcare, which creates a different context for considering nature prescriptions.

It was clear throughout our research, however, that Indigenous Peoples should not be left out of the clear ongoing health benefits of Nature as they have been interacting with Nature for their health and wellbeing for millennia; however, engagement within Nature may be better premised within *a way of life* approach with an expansion on what constitutes a Nature activity in the Westernized sense to be inclusive of cultural activities like hunting, fishing, and food foraging as examples. The connections to nature prescription programs may generally seem to be potentially irrelevant and culturally incongruent, which was highlighted within our research findings; however, given the current overall lack of appropriate health funding for *on-the-Land* activities for Indigenous Peoples, nature prescription programs may provide opportunities for funding and resources to allow communities to continue interacting with Nature in their own self-determined way. Land-based cultural activities, for example, were stated within our findings to be situated within the collective community activities that have been a part of Indigenous lived experiences for generations (e.g., hiring community members to take people out on the Land for rest and wellbeing purposes).

Given the Land-based orientation most common within Indigenous community settings, a reconsideration of the nature prescription terminology was also stated to be warranted within Indigenous patient care settings with a focus on *Land-based* or *on-the-Land* health promotion activities that are again culturally situated. Despite many physicians, in particular, noting clear discomfort with prescribing nature to Indigenous patients, many were already having verbal conversations with Indigenous patients on being out on the Land within the context of their health and wellbeing without issue. Given this, the definition of a nature prescription in the context of Indigenous Peoples may need to be expanded outside of having a written prescription pad, but instead seeing nature prescriptions through verbal health promotion approaches that amplify existing Indigenous knowledge on the health benefits of being out on the Land for improved health outcomes.

With this, Nature prescription educational materials could therefore be developed to support physicians themselves in understanding how to amplify and support Indigenous patients in their longstanding Land-based activities through culturally safe approaches as opposed to physicians educating or giving prescription directives to Indigenous patients. As one Indigenous Elder participant aptly stated, “we can provide that prescription to those doctors”. This comment raises the important notion that instead of health systems considering how to prescribe Nature, perhaps it is the health system that needs the *prescription* of listening and learning from Indigenous Peoples in regard to Land-based healing approaches. Regardless, given our findings as well as the increasing uptake of nature prescriptions within health systems, there is a substantial need for more consideration to be given to the mechanics and process of operationalizing nature prescription programs where Indigenous Peoples reside.

### Limitations

Our study was exploratory, with a small sample size, in a northern Canadian geographic context. Given that many of the physicians interviewed are not originally from the NWT (i.e., moved to the NWT region to practice medicine), we sense, however, that there may be insights relevant to consider for other contexts where Indigenous Peoples reside. Regardless, Indigenous Peoples are not a homogenous group so the views shared from this region may not necessarily be completely reflected in other Indigenous contexts. Despite this, colonization is a common experience for many Indigenous Peoples, which is reflected in some of the sentiments shared from this region. As noted, however, this is the first exploratory research inquiry (to the best of our knowledge) into nature prescribing within the Indigenous patient context; therefore, further investigation is warranted to ensure that any potential outside implementation of nature prescribing is culturally safe and considered within the context of Indigenous self-determination.

## 5. Conclusions

Despite the perceived and demonstrated health benefits of being in nature, nature prescriptions themselves within the context of Indigenous Peoples in Canada and globally have received little attention. With greater awareness of the health benefits of nature and the expanding nature prescription programs in Canada and abroad, our research identified key relevance and implementation considerations when serving Indigenous communities. These considerations include the need to question the relevance of nature prescriptions within Indigenous communities generally; the need for culturally appropriate and context-specific terminology to be used for framing nature prescriptions where appropriate; physician awareness of any cultural considerations when engaging nature prescriptions within Indigenous community settings; and attunement to the kinds of activities that may be considered *on-the-Land* activities to support Indigenous wellbeing outside of the more commonly understood nature prescription activities in a Westernized context.

## Data Availability

The data that support the findings of the interview portion of this study are available from the corresponding author (N.R.) upon reasonable request. Due to Indigenous ethical considerations and Indigenous data sovereignty, any data and materials associated with the sharing circle portion of the research will not be available unless additional relevant Indigenous ethics agreements are in place with the Elders.

## Supplementary Materials

The following supporting information can be downloaded at: https://www.mdpi.com/article/10.3390/ijerph21060806/s1, S1 file: Indigenous Elders Focus Group Discussion Guide, Physician Interview Discussion Guide, Verbal Consent Form—Focus Groups, Verbal Consent Form—Interview.
