# Supplementary material for: Nature Prescriptions and Indigenous Peoples: A Qualitative Inquiry in the Northwest Territories, Canada
Source: Int J Environ Res Public Health. 2024 Jun 20;21(6):806. doi: 10.3390/ijerph21060806 (PMC11203734; doi:10.3390/ijerph21060806)
Supplement: Supplementary file 1 [file ijerph-21-00806-s001.zip › ijerph-3063763-supplementary.pdf]

Supplementary Materials

Title: Nature Prescriptions and Indigenous Peoples: A qualitative inquiry in the Northwest Territories, Canada

Table of Contents

Supp 1A- Indigenous Elders Focus Group Discussion Guide Pg. 1

Supp 2A- Physician Interview Discussion Guide Pg. 2

Supp 3A- Verbal Consent Form-Focus Groups Pg. 2

Supp 4A- Verbal Consent Form-Interview Pg. 3

Supp 1A- Indigenous Elders Focus Group Discussion Guide for Nature Prescriptions

Topics to be explored

Question 1 preamble: Right now, some doctors in the southern provinces have started up a movement to have doctors and other health care providers prescribe nature to patients to improve their health and wellbeing. They call this 'nature prescriptions' or 'park prescriptions'. The nature prescription given to a patient might involve going for a walk in nature, or just spending time in nature which has shown to have mental and physical health benefits for individuals.

Question 1: How would you feel if your doctor or nurse gave you a nature prescription?

Additional Prompts:

a. Can you see doctors and nurses prescribing nature to patients in the NWT? Why or why not?

b. Are there situations where you could see nature prescriptions being helpful to patients in the NWT?

c. Are there any situations where you could see nature prescriptions causing problems or challenges in the delivery of medical care in the NWT? Why do you think this is the case?

d. What might make it difficult for doctors and nurses in your region to use nature prescriptions?

Question 2: How do you feel about the name 'nature prescription'?

Additional Prompts:

a. Do you think the name 'nature prescriptions' makes sense?

b. Do you think other names for nature prescriptions should be considered in the context of the NWT, and if so, what might be some alternative names you would suggest?

c. How do you feel about doctors and nurses utilizing 'on the land' prescriptions instead?

d. What might be the benefits or challenges with doctors and nurses using the name ‘on the land’ prescriptions instead of other possible names?

Question 3 preamble: To wrap up our discussion today,

Question 3: Is there anything else anyone would like to add or comment on?

Supp 2A- Physician Interview Discussion Guide for Nature Prescriptions

Topics to be explored

Views on specific prescribing practices

Question 1 preamble: Right now, some physicians in the southern provinces have started up a movement to have doctors and other health care providers prescribe nature to patients to improve their health and wellbeing. They call this ‘nature prescriptions’ or ‘park prescriptions’. The nature prescription given to a patient might involve going for a walk in nature, or just spending time in nature which has shown to have mental and physical health benefits for individuals.

Question 1: Would you feel comfortable now giving patients a nature prescription? Why or why not?

Additional Prompts:

a. Can you see physicians prescribing nature to patients in the NWT widely? Why or why not?
b. Have you prescribed nature in any way to your patients already, and what has your experience been like giving these prescriptions if you have? Have you heard of colleagues giving these prescriptions and what experiences have they had doing this?
c. Are there situations where you could see nature prescriptions being helpful to patients in the NWT?

d. Are there any situations where you could see nature prescriptions causing problems or challenges in the delivery of medical care in the NWT? Why do you think this is the case?

e. What might make it difficult for physicians in your region to use nature prescriptions?

Question 2 preamble: To wrap up our discussion today,

Question 2: Is there anything else you would like to add or comment on?

Supp 3A- Verbal Consent Form-Focus Groups

VERBAL CONSENT FORM- FOCUS GROUPS

Prescribing planetary health in a circumpolar health region

If you are happy to take part in this study and to give your oral consent, I will read out the Informed Consent Form that you have received a copy of, and would like you to answer ‘yes’ or ‘no’ to each of the questions.

Tick the box if confirmed

☐

1. I confirm that I have read and understand the participant information sheet version ‘1, 11th July 2021’, and that I have had the opportunity to ask questions which have been answered to my satisfaction.

2. I understand that my participation is voluntary and that I am free to withdraw at any stage without giving reasons and without my legal rights being affected.

3. I understand that all my details will be kept confidential, that no personal information will appear on any reports or documents and only a unique ID number will be used.

4. I understand that the focus group will be audio-recorded and that the focus group will be transcribed by an independent person to have a written record.

5. I understand that data collected during the study may be looked at by individuals from the research team from the University of Oxford or from regulatory authorities, where it is relevant to my taking part in this research.

6. I agree to take part in this research study.

______________________	______________________
Name of Participant	*Date*

______________________	______________________	______________________
Name of Person taking Consent (Print)	*Date*	*Signature*

No signature obtained from the participant as verbal consent taken by telephone/video conference

**1 copy for participant; 1 copy for researcher*

Supp 4A- Verbal Consent Form-Interview

VERBAL CONSENT FORM- INTERVIEWS

Prescribing planetary health in a circumpolar health region

If you are happy to take part in this study and to give your oral consent, I will read out the Informed Consent Form that you have received a copy of, and would like you to answer ‘yes’ or ‘no’ to each of the questions.

Tick the box if confirmed

7. I confirm that I have read and understand the participant information sheet version ‘1, 11th July 2021’, and that I have had the opportunity to ask questions which have been answered to my satisfaction.

8. I understand that my participation is voluntary and that I am free to withdraw at any stage without giving reasons and without my legal rights being affected.

9. I understand that all my details will be kept confidential, that no personal information will appear on any reports or documents and only a unique ID number will be used.

10. I understand that the interview will be audio-recorded and that the interview will be transcribed by an independent person to have a written record.

11. I understand that data collected during the study may be looked at by individuals from the research team from the University of Oxford or from regulatory authorities, where it is relevant to my taking part in this research.

12. I agree to take part in this research study.

______________________	______________________
Name of Participant	*Date*

______________________	______________________	______________________
Name of Person taking Consent (Print)	*Date*	*Signature*

No signature obtained from the participant as verbal consent taken by telephone/video conference

**1 copy for participant; 1 copy for researcher*
